# Tourniquet cuff pressure during blood flow restriction exercise

**DOI:** 10.3389/fspor.2025.1582387

**Published:** 2025-09-02

**Authors:** Patrick Swain, James McEwen, Tom Lai, Luke Hughes

**Affiliations:** ^1^ Aerospace Medicine and Rehabilitation Laboratory, Department of Sport, Exercise and Rehabilitation, Faculty of Health and Life Sciences, Northumbria University, Newcastle upon Tyne, United Kingdom; ^2^ Western Clinical Engineering Ltd., Vancouver, BC, Canada; ^3^ School of Biomedical Engineering and Departments of Orthopedics and Electrical and Computer Engineering, University of British Columbia, Vancouver, BC, Canada

**Keywords:** blood flow restriction, surgical-grade, tourniquet, pressure, autoregulation, exercise

## Abstract

**Background:**

The present study examined how well different blood flow restriction (BFR) devices deliver the prescribed tourniquet cuff pressure.

**Methods:**

Fifteen participants completed four BFR exercise sessions, each with a different BFR device [Delfi Personalized Tourniquet System (PTS) for BFR, Saga, SmartCuffs, and Suji], comprising four sets of unilateral leg press (30-15-15-15 repetitions) against resistance bands with 30-second rest periods. The tourniquet cuff was secured proximally on the exercising leg, and the target pressure was set to 80% limb occlusion pressure (LOP), as measured by the device, applied continuously throughout the exercise/rest periods. Tourniquet cuff pressure was sampled at 100 Hz via a pressure transducer.

**Results:**

Despite prescribing tourniquet cuff pressure at 80% LOP, the actual pressure can vary substantially and be inconsistent between individuals depending on the BFR device used. During the exercise periods, the median percentage of time pressure was within ±10% the target pressure was 95% (Delfi PTS for BFR), 25% (Saga), 26% (SmartCuffs), and 34% (Suji). During the rest periods, the median percentage of time pressure was within ±5% the target pressure was 99% (Delfi PTS for BFR), and 0% for the Saga, SmartCuffs, and Suji BFR devices. Tourniquet cuff pressure during BFR exercise behaves in a wave-like manner characterised by cyclical pressure peaks and valleys. The magnitude of pressure peaks and valleys was: Delfi PTS for BFR (89 ± 2% and 72 ± 3% LOP), Saga (79 ± 9 and 58 ± 7% LOP), SmartCuffs (79 ± 9% and 61 ± 7% LOP), and Suji (90 ± 15 and 65 ± 10% LOP). In several cases, participants experienced tourniquet pressures >100% LOP using the Saga, SmartCuffs, and Suji BFR devices, for up to ∼30%–55% of the exercise set duration. A progressive loss of pressure occurred throughout the BFR application period by an average of 2–4 mmHg·min^−1^ (∼1%–2% LOP·min^−1^) in the Saga, SmartCuffs, and Suji BFR devices, whilst the Delfi PTS for BFR exhibited trivial pressure drifts.

**Conclusion:**

Differences between the actual and prescribed tourniquet cuff pressure can highly depend on the BFR device employed. The selection of the BFR apparatus is thus important to delivering the prescribed tourniquet cuff pressure to allow for standardisation of the relative occlusion pressure between users.

## Introduction

Exercising under blood flow restriction (BFR) involves the application of a tourniquet to the proximal portion of the limb(s) with the goal of partially restricting distal arterial blood flow and fully occluding venous outflow (1). Tourniquet cuff pressure plays a fundamental role in mediating limb blood flow, tissue perfusion, and haemodynamic responses to BFR exercise (2–4). Historically in BFR tourniquet use, the pressure to which the tourniquet cuff is inflated (i.e., the target pressure) to has involved non-individualised methods such as arbitrary fixed pressures (e.g., 200 mmHg) or as a percentage of systolic blood pressure (SBP). While the latter is technically individualised, it has significant limitations compared to the gold-standard method of using limb occlusion pressure (LOP). Blood pressure cuffs are designed for blood pressure measurement, requiring a specific bladder width to limb circumference ratio, and use pneumatic bladders that do not fully encircle the limb, unlike tourniquet cuffs which are specifically engineered to provide uniform circumferential pressure for BFR applications. The type of tourniquet cuff used (e.g., design, width, and material) can also significantly impact LOP (5). Limb occlusion pressure in the arm and leg can differ substantially from SBP and individuals with the same SBP can have different LOPs, and vice versa (6). This variability makes BFR applications using SBP-based pressure prescription inappropriate which can affect BFR safety, efficacy, and tolerability (1, 7, 8). Prescribing tourniquet cuff pressure on an individual basis at 40%–80% of the minimum pressure required to fully occlude arterial blood flow beneath the cuff, termed LOP, is now the recommended method for standardising BFR applications (1, 7).

A fundamental tenet of BFR applications includes the assumption that the pressure within the tourniquet cuff is maintained and regulated at, or close to, the target pressure. It must be acknowledged, however, that few have set forth to verify such an assumption. Maintenance of the target pressure reflects the absence of any pressure gain/loss across time within the tourniquet system during a passive application period (i.e., at rest). Regulation of the target pressure reflects the active monitoring of pressure within the system and adapting the pneumatic response of the system to continuously attenuate differences between the actual and target pressure. Some variation in tourniquet cuff pressure can be expected due to the dynamic and transient nature of BFR exercise; a primary factor implicated for tourniquet cuff pressure variability includes changes in muscle circumference during contraction/relaxation phases causing pressure spikes/troughs (9). It is, however, unlikely that every BFR device contains equivalent pneumatic control systems and, therefore, raises the question whether different BFR devices vary in their ability to maintain and regulate tourniquet cuff pressure at the target pressure. Hughes et al. (10) examined tourniquet cuff pressure (sampled at 100 Hz) during BFR leg pressure exercise using five commercially available BFR devices and demonstrated that in four of the five devices the percentage of time pressure remained within ±15 mmHg the target pressure for any given second was only ∼40%–60% (standard deviation: ±24%–36%), revealing meaningful variation in tourniquet cuff pressure within and across BFR devices.

Beyond the initial investigation of Hughes et al. (10), the specific characteristics of tourniquet cuff pressure variability, such as pressure distributions, pressure peak and valley magnitudes, pressure drift, and instances of pressures exceeding 100% LOP, remain unexplored, despite the foundational importance of tourniquet cuff pressure in BFR applications.

The present report, therefore, utilises the dataset originally collected by Hughes et al. (10) to conduct a more detailed characterisation of tourniquet cuff pressure behaviour between commercially available BFR devices (Delfi Personalized Tourniquet System for BFR, Saga, SmartCuffs, and Suji). This extended analysis offers new, pioneering insight into pneumatic tourniquet cuff performance characteristics including: (1) the percentage of time tourniquet cuff pressure remained within fixed windows around the target pressure (e.g., ±2.5%, ±5%, ±10%, ±15%, ±20%), (2) the distribution of pressure values during exercise and resting BFR applications, (3) the magnitude of pressure peak and valley during exercise, (4) the percentage of time pressure exceeded 100% LOP, (5) the presence and direction of pressure drift (e.g., a gradual gain/loss of pressure over time). Through this re-analysis, we offer an enhanced understanding of the tourniquet cuff pressure waveform across different BFR devices and quantity the actual pressures being applied as a percentage of LOP throughout a standard BFR application.

## Materials and methods

The present report uses raw data by Hughes et al. (10) to which full study details can be found in the original publication, summarised briefly herein. The present analysis does not duplicate outcome measures from our previous work. The B Strong Training System (B Strong, Utah, USA) was investigated in the original study, however, was not examined in the present study for this device did not allow for the automatic measurement of LOP and instead had to be set at an absolute pressure (300 mmHg) in accordance with the manufacturer's instructions. As LOP reflects the minimum pressure required to fully occlude arterial blood flow beneath and using a specific cuff and is influenced by cuff design (e.g., width and material), comparing absolute changes in pressure between devices may not reflect the same change in pressure when expressed as a percentage of LOP.

### Participants

Fifteen healthy individuals (age: 38 ± 14 years; bodyweight: 71 ± 16 kg; height: 1.70 ± 0.1 m; male: female = 8:7) participated in the study. No participant was reported to have any contraindications to tourniquet use, were recreationally active, and free from cardiovascular, pulmonary, and metabolic diseases, and musculoskeletal injuries in the preceding 12-months. Participants were instructed to refrain from strenuous exercise, caffeine, and alcohol in the 24 h period prior to testing sessions. The study protocol was approved by the Northumbria University Ethics Board and all participants provided written informed consent.

### Experimental design and protocol

Participants completed five sessions of single-leg horizontal leg press with BFR across three visits. Each exercise session was performed with a different BFR system in a randomised order. The original study examined five BFR devices. Visits one and two included two BFR sessions and visit three included one BFR session. A 10 min rest period separated sessions performed in the same visit. A minimum of 12 h separated each visit. The exercising limb was randomised for session one and alternated for each successive session. Each BFR device was used on one limb only within each session. Readers are referred to the original study for full exercise protocol details (10). Briefly, participants laid on a horizontal leg press machine (Weider Ultimate Body Work Exercise Machine WEBE15911) with three resistance bands attached. Resistance bands were employed to accommodate the design of the leg press machine. In addition, it was believed that the primary driver of tourniquet cuff pressure fluctuations during BFR exercise was caused by changes in circumference of the cuffed limb that occur throughout the range of motion of the exercise, rather than the exercise load. Six familiarisation leg press repetitions were performed followed by a 3 min rest. Four sets of BFR leg press exercise were then performed using a 30-15-15-15 repetition scheme with 2 s concentric and eccentric phases for each repetition (using a metronome) with 30 s inter-set passive rest periods. In each session, LOP was measured by the BFR device while the participant was in the same body position as the leg press exercise (i.e., supine horizontal), within 60 s prior to the exercise. The target/prescribed tourniquet cuff pressure was set to 80% of the measured LOP throughout the exercise and rest periods (11). The absolute target pressure (i.e., 80% LOP) for each system was as follows: 155 ± 15 mmHg (Delfi Personalized Tourniquet System for BFR), 191 ± 34 mmHg (Saga), 157 ± 13 (SmartCuffs), and 135 ± 11 (Suji). Variations in limb occlusion pressure (LOP) readings, expressed in absolute pressure units (mmHg), across different blood flow restriction (BFR) devices can be largely attributed to differences in device characteristics, such as tourniquet cuff shape (e.g., cylindrical vs. contoured), bladder width (narrow vs. wide), bladder length (whether it fully or partially encircles the limb), and the materials used in the cuff or bladder (e.g., presence of stiffeners or elastic components) (12). The validity of the BFR device's LOP measurement also contributes to LOP variability.

### BFR systems

Four commercially available BFR systems (i.e., pressure control device and corresponding tourniquet cuff) were examined: (1) Delfi Personalized Tourniquet System (PTS) for BFR (Delfi Medical Innovations, Vancouver, Canada), (2) Saga—The BFR Cuffs (Saga Fitness, Newstead, Australia), SmartTools—SmartCuffs 3.0 (STP) (Smart Tools, Ohio, USA), and Suji Generation 1.0 (Suji, Scotland, UK). The BFR device properties are reported in Supplementary Table S1 in accordance with BFR instrument reporting recommendations (12). The reporting item “Validity and Reliability of Limb Occlusion Pressure Measurement” includes references to studies investigating the validity and/or reliability of the LOP measurement for each BFR device as provided by the manufacturer upon email request or identified in literature searches. Cuff sizes were selected for the participant's limb based on the manufacturer's recommendations. Limb occlusion pressure was determined following the manufacturer's instructions. Modifications to the BFR systems to enable the measurement of tourniquet cuff pressure are quoted from the original publication of Hughes et al. (10): “The BFR systems were modified with a Y-connection to connect to a pressure acquisition module, which consisted of a MPX5100 pressure transducer and peripheral circuitry to communicate to a LabVIEW data acquisition program via USB. The pressure acquisition module introduced approximately 0.3% of the total volume in the pneumatic system. The pressure acquisition module does not have any active components that could interfere with the cuff or affect the delivery of pressure to the cuff. The data acquisition module was calibrated using a calibrated 3D Instruments DTG-5000 digital test gauge. For the B strong, Delfi PTS for BFR and SmartCuff devices, no physical modification was required; the “Y” connection from the pressure sensor is simply introduced into the pneumatic system via existing connectors. For the Suji system, positive locking connectors were added to the tubing to enable the “Y” connector from the pressure sensor to be introduced between the two connectors. For the Saga system, the Saga instrument was separated from the Saga cuff and tubings and connectors were added to the cuff and to the instrument. The “Y” connector from the pressure sensor was then introduced between the cuff and the instrument at the sensing channel”.

### Data analysis

Raw pressure data were analysed using a custom script written in MATLAB (Version R2024b, MathWorks, Massachusetts, US). The original data sets and code are publicly available (see Data Availability Statement). The pressure signal was of a high quality and no digital filter was applied; raw pressure data can be accessed in the Data Availability Statement. The percentage of time tourniquet cuff pressure was within ±2.5%, ±5%, ±10%, ±15%, and ±20% of the target pressure (i.e., 80% of the measured LOP) was calculated across the exercise and rest periods for each BFR device. For example, ±5% of the target pressure is equal to 75%–85% LOP. The distribution of tourniquet cuff pressures was determined using the non-parametric kernel probability distribution function (bandwidth = 1) and represented with ridgeline plots (13). Kernel distributions were determined during the “dynamic” (exercise) and “passive” (rest) BFR periods. Each tourniquet cuff pressure peak and valley were automatically detected in MATLAB. The mean pressure peaks and valleys were computed across the BFR application period for each participant. The percentage of time during which tourniquet cuff pressure exceeded 100% LOP was determined for each set of exercise. Pressure drifts across the entire BFR application period, and independently for pressure peaks and valleys, were determined via linear regression with respect to time.

### Statistics

The Statistical Package for Social Sciences (Version 29.0.1.0, IBM Corp, Chicago, Illinois) was used for statistical analysis. No *a priori* sample size calculation was performed in the original study. However, a sample size of *n* = 15 is sufficient to detect effect sizes ranging from moderate to large (Cohen's *f* = 0.25–0.40) in a within-subjects (repeated-measures) ANOVA design, assuming a typical correlation among repeated measures (*r* = 0.5). Statistical significance was set *a priori* at *p* < 0.05. The Friedman Test compared differences in the percentage of time tourniquet cuff pressure was within specific windows of the target pressure (±2.5%, ±5%, ±10%, ±15%, and ±20%) between BFR devices as this data did not conform to a normal distribution. Significant main effects were further examined using simple *post-hoc* pairwise comparisons using Wilcoxon signed-rank tests with adjusted *p*-values (Bonferroni method). Non-parametric data are presented using the median and interquartile range (IQR). A one-way repeated measures analysis of variance (ANOVA) compared differences in the mean tourniquet cuff pressure peaks and valleys, and pressure drifts across the BFR application period between each BFR device. Data were verified for normality (Shapiro–Wilks test and skewness and kurtosis) and sphericity (Mauchly's Test of Sphericity). Simple *post-hoc* pairwise comparisons were examined with Bonferroni adjustments. Effect sizes were computed using partial eta squared (*η*_p_^2^). Data are presented as mean ± standard deviation unless otherwise specified.

## Results

### Pressure distribution

The distribution of pressures measured within the tourniquet cuff during all BFR exercise and rest periods are displayed in Figure 1. Pressure distributions for each exercise and rest period for every participant and BFR device can be found in the Supplementary Figures S1–S8.

**Figure 1:**
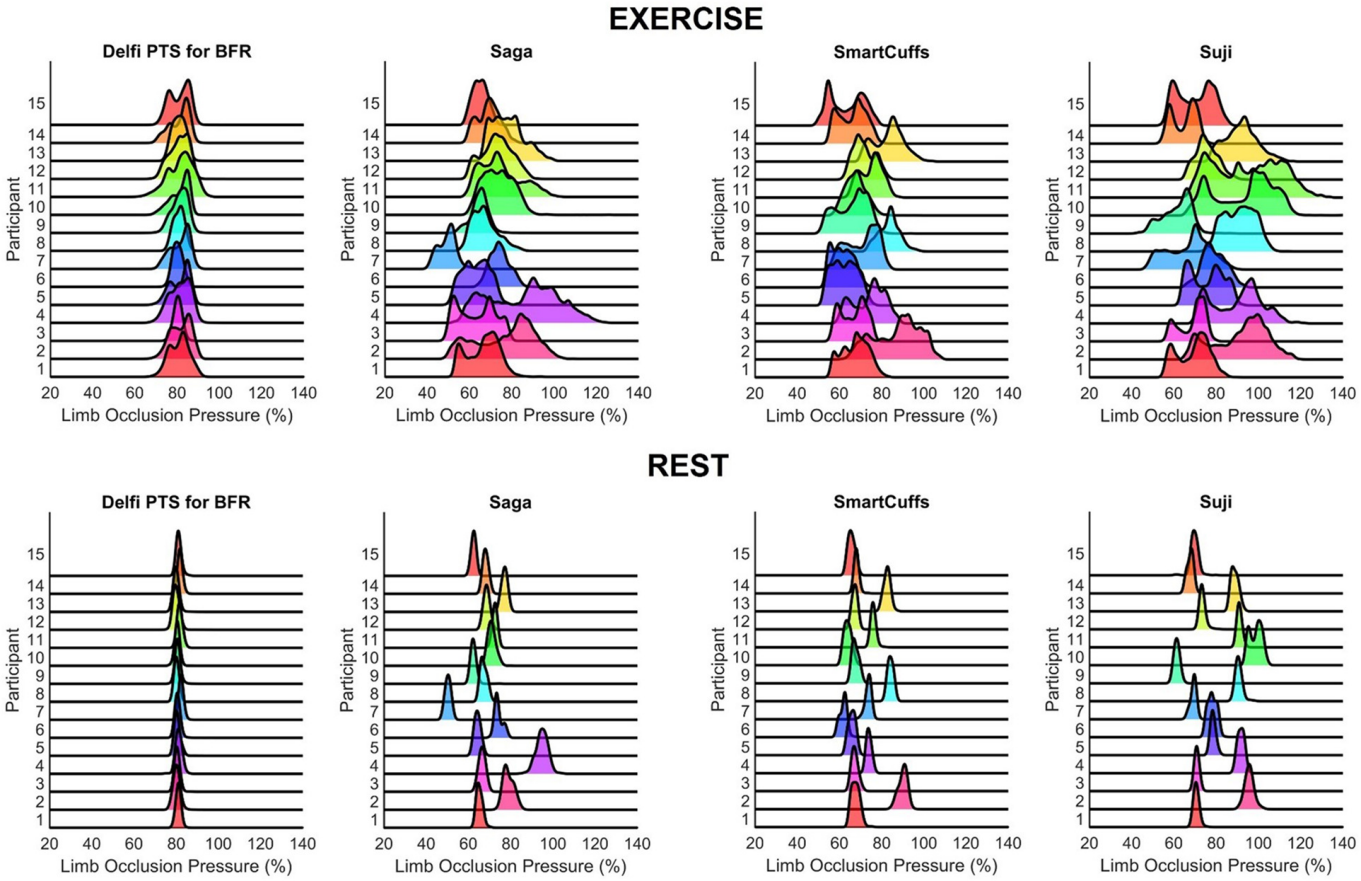
Distribution of tourniquet cuff pressure throughout the BFR exercise (top figures) and rest (bottom figures) periods (*n* = 15).

### Percentage of time tourniquet cuff pressure was within ±2.5%, ±5%, ±10%, ±15%, and ±20% the target pressure during exercise and rest

Table 1 displays the percentage of time tourniquet cuff pressure was within a fixed range around the target pressure (e.g., ±5% = 75%–85% LOP) for each BFR device during the exercise and rest periods. During the exercise periods there was a statistically significant difference in the time tourniquet cuff pressure was kept within each pressure window around the target pressure (all *p* < 0.001). Tourniquet cuff pressure during the exercise periods was kept within each target pressure window for a significantly greater percentage of time for the Delfi PTS for BFR compared to the Saga, SmartCuffs, and Suji BFR devices (all *p* < 0.01). No significant pairwise differences were found between the Saga, SmartCuffs, and Suji BFR devices. Individual data and pairwise comparisons can be found in the Supplementary Excel File (sheet: “Pressure Windows Exercise”).

**Table 1:** Percentage of time tourniquet cuff pressure was within ±2.5%, ±5%, ±10%, ±15%, and ±20% of the target pressure (*n* = 15).

Target pressure window (% LOP)	Percentage of time within the pressure range				Friedman test	
	Delfi PTS BFR	Saga	SmartCuffs (STP)	Suji	*χ* ^2^	*p*
Exercise periods						
77.5–82.5% (±2.5%)	24% (20–39%)	4% (1–13%)	3% (1–16%)	8% (5–15%)	22.600	<0.001
75–85% (±5%)	49% (47–74%)	10% (4–27%)	8% (4–30%)	16% (11–27%)	24.760	<0.001
70–90% (±10%)	95% (93–99%)	25% (14–52%)	26% (20–62%)	34% (30–44%)	27.960	<0.001
65–95% (±15%)	100% (100–100%)	48% (32–72%)	50% (45–75%)	53% (42–64%)	27.720	<0.001
60–100% (±20%)	100% (100–100%)	66% (58–86%)	76% (62–94%)	68% (57–74%)	23.400	<0.001
Rest periods						
77.5–82.5% (±2.5%)	89% (78–94%)	0% (0–0%)	0% (0–0%)	0% (0–0%)	34.109	<0.001
75–85% (±5%)	99% (98–99%)	0% (0–0%)	0% (0–2%)	0% (0–1%)	28.907	<0.001
70–90% (±10%)	100% (100–100%)	0% (0–62%)	1% (0–97%)	1% (0–33%)	16.714	<0.001
65–95% (±15%)	100% (100–100%)	34% (2–100%)	41% (19–100%)	85% (56–99%)	9.783	0.021
60–100% (±20%)	100% (100–100%)	100% (60–100%)	100% (92–100%)	100% (98–100%)	8.259	0.041

Note: Data are median (quartile 1—quartile 3).

During the rest periods, there was a statistically significant difference in the time tourniquet cuff pressure was kept within each pressure window around the target pressure (all *p* < 0.05). Tourniquet cuff pressure during the rest periods was kept within each target pressure window for a significantly greater percentage of time for the Delfi PTS for BFR compared to the Saga, SmartCuffs, and Suji BFR devices (all *p* < 0.01), except for the ±20% window (i.e., 60%–100% LOP) in which no significant pairwise differences were found. No significant differences were found between the Saga, SmartCuffs, and Suji BFR devices. Individual data and pairwise comparisons can be found in the Supplementary Excel File (sheet: “Pressure Windows Rest”).

### Pressure peaks and valleys

**Figure 2:**
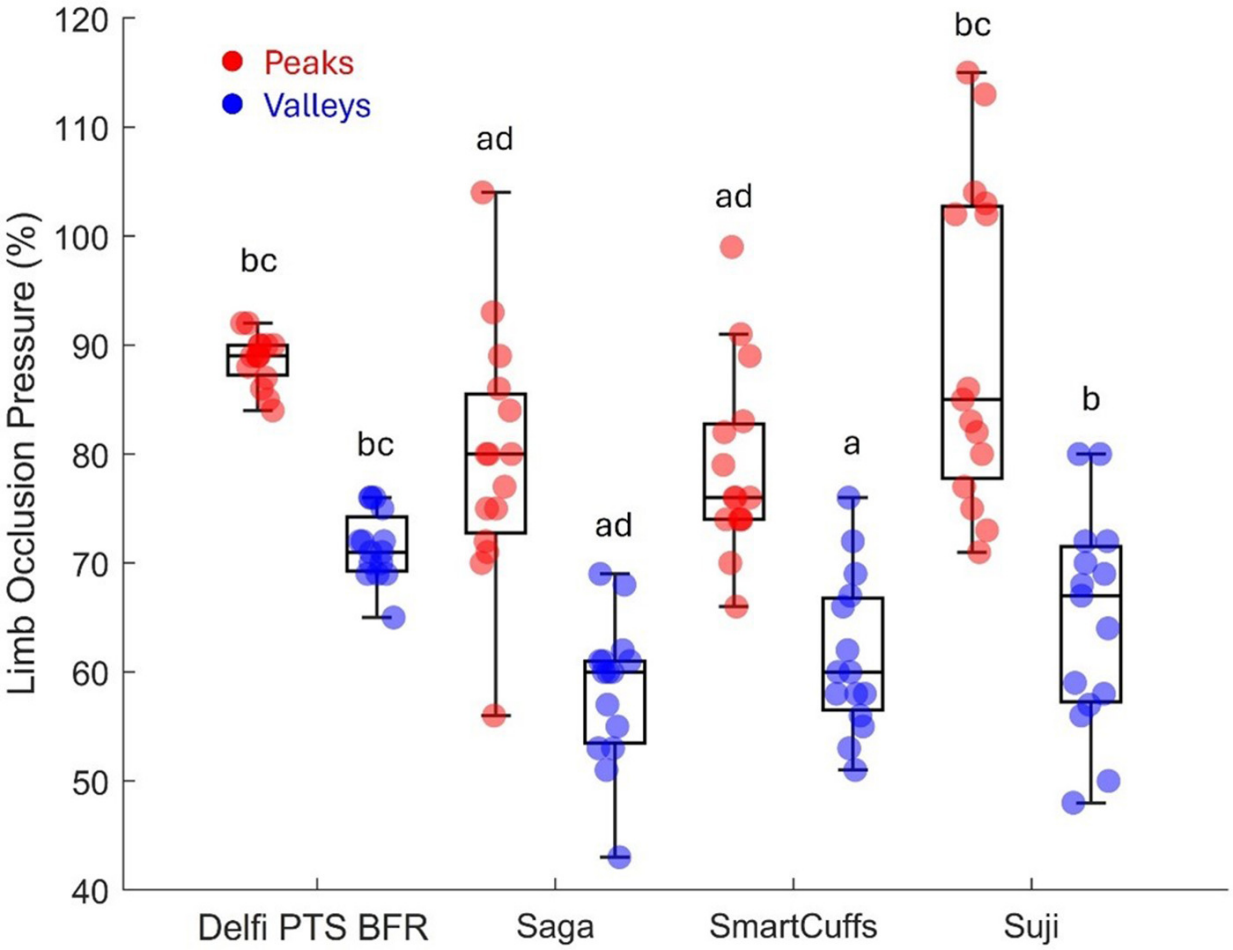
Pressure peaks and valleys (*n* = 15). Boxplots display median (middle box line), interquartile range (box boarders), and minima and maxima values (error bars). Letters represent significant (*p* < 0.05) pairwise differences between BFR devices: Delfi Personalized Tourniquet System [PTS] for BFR **(a)**, Saga **(b)**, SmartCuffs **(c)**, and Suji **(d)**.

Table 2 and Figure 2 display tourniquet cuff pressure peaks and valleys that occurred during the exercise period. Tourniquet cuff pressure peaks and valleys were significantly different between BFR devices (both *p* < 0.001). Individual data and pairwise comparisons can be found in the Supplementary Excel File (sheet: “Pressure Peaks” and “Pressure Valleys”).

**Table 2:** Tourniquet cuff pressure peaks and valleys (*n* = 15).

Outcome	Limb occlusion pressure (%)				ANOVA		
	Delfi PTS BFR	Saga	SmartCuffs	Suji	*F*	*p*	*η*_p_^2^
Pressure Peaks	89 ± 2	79 ± 11	79 ± 9	90 ± 15	8.086	<0.001	0.366
Pressure Valleys	72 ± 3	58 ± 7	61 ± 7	65 ± 10	15.815	<0.001	0.530

Abbreviations: *η*_p_^2^, partial eta squared.

### Pressures exceeding limb occlusion pressure

**Figure 3:**
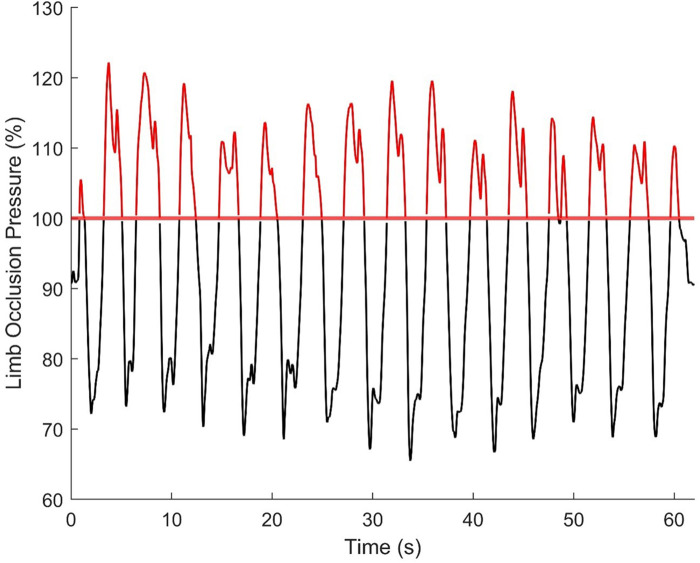
Example of tourniquet cuff pressure behaving in a wave-like manner during BFR exercise (15 leg press repetitions) and exceeding limb occlusion pressure. Data are from participant 11, exercise set 2, using the Suji BFR device and reflects one of the highest percentages of time (44%) pressure was beyond 100% limb occlusion pressure.

Table 3 displays the percentage of time tourniquet cuff pressure exceeded 100% LOP for each BFR device and participant. Figure 3 illustrates tourniquet cuff pressure exceeding LOP. Tourniquet cuff pressures >100% LOP were variable between BFR devices and participants. Only the Delfi PTS for BFR was found to not have tourniquet cuff pressures >100% LOP for any participant.

**Table 3:** Percentage of time tourniquet cuff pressure exceeded 100% limb occlusion pressure (LOP).

Participant	Percentage of time pressure was above 100% LOP (%)															
	Delfi PTS BFR				Saga				SmartCuffs				Suji			
	Exercise Set				Exercise Set				Exercise Set				Exercise Set			
	1	2	3	4	1	2	3	4	1	2	3	4	1	2	3	4
1	0	0	0	0	0	0	0	0	0	0	0	0	0	0	0	0
2	0	0	0	0	**3**	0	0	0	**30**	0	**1**	0	**54**	**28**	**7**	0
3	0	0	0	0	0	0	0	0	0	0	0	0	0	0	0	0
4	0	0	0	0	**43**	**17**	**2**	0	0	0	0	0	**46**	**2**	**2**	**4**
5	0	0	0	0	0	0	0	0	0	0	0	0	0	0	0	0
6	0	0	0	0	0	0	0	0	0	0	0	0	0	0	0	0
7	0	0	0	0	0	0	0	0	0	0	0	0	0	0	0	0
8	0	0	0	0	0	0	0	0	0	0	0	0	**28**	**2**	**1**	**1**
9	0	0	0	0	0	0	0	0	0	0	0	0	0	0	0	0
10	0	0	0	0	0	0	0	0	0	0	0	0	**53**	**39**	**31**	**31**
11	0	0	0	0	**1**	0	0	0	0	0	0	0	**55**	**44**	**41**	**19**
12	0	0	0	0	0	0	0	0	0	0	0	0	0	0	0	0
13	0	0	0	0	0	0	0	0	**2**	0	0	0	**33**	**6**	0	0
14	0	0	0	0	0	0	0	0	0	0	0	0	0	0	0	0
15	0	0	0	0	0	0	0	0	0	0	0	0	0	0	0	0

Bold values highlight occurrences of pressures exceeding 100% LOP. Note: Percentages are rounded to the nearest integer.

### Pressure drift

**Figure 4:**
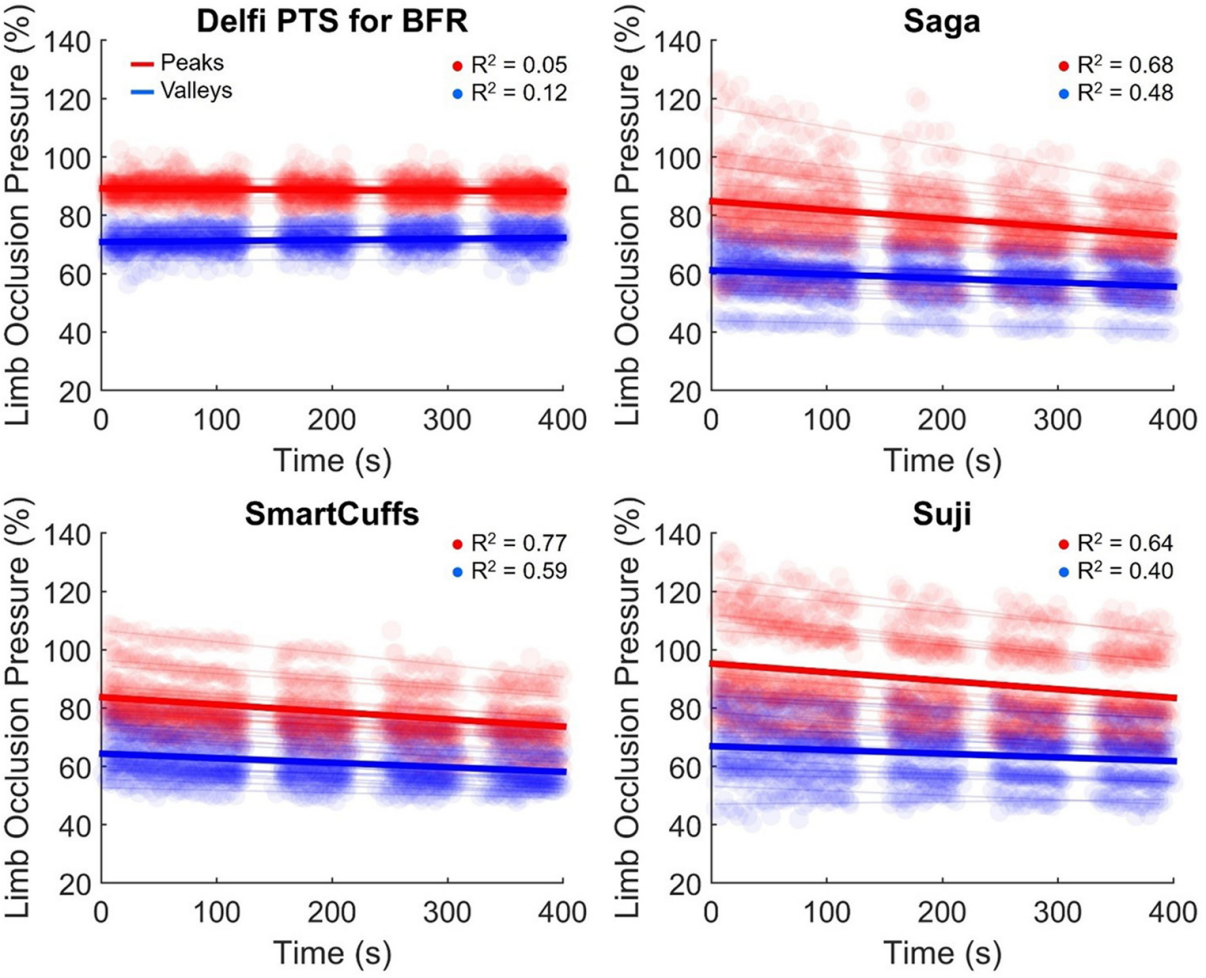
Drift in pressure peaks (red) and valleys (blue) during the BFR application period. Semi-transparent scatter markers represent individual pressure peaks and valleys for each participant. Semi-transparent lines represent the average peak and valley linear regression slope for each participant. The average cohort drift in peak and valley pressures are denoted by respective red and blue bold regression lines (*n* = 15).

Table 4 displays the change in tourniquet cuff pressure with respect to time. Peak and valley pressure drifts are presented in Figure 4. The Saga, SmartCuffs, and Suji BFR devices progressively lost pressure throughout the BFR application period by an average of ∼2–3 mmHg·min^−1^ (∼1%–2% LOP·min^−1^), whereas the Delfi PTS for BFR displayed trivial changes. The relationship between pressure and time was weak for all devices (*R*^2^ = 0.00–0.22) due to the wave-like nature of tourniquet cuff pressure. Individual data and pairwise comparisons can be found in the Supplementary Excel File (sheet: “Drift %LOP” and “Drift mmHg”).

**Table 4:** Tourniquet cuff pressure drifts (*n* = 15).

Outcome	Unit	Delfi PTS BFR	Saga	SmartCuffs	Suji	ANOVA		
						F	*p*	*η*_p_^2^
Mean pressure drift	mmHg·min^−1^	−0.1 ± 0.2	−3.2 ± 1.3	−2.5 ± 0.7	−2.2 ± 0.9	48.211	<0.001	0.736
	%LOP·min^−1^	−0.0 ± 0.1	−1.6 ± 0.7	−1.3 ± 0.4	−1.1 ± 0.5	47.707	<0.001	0.773
	*R* ^2^	0.00 ± 0.00	0.19 ± 0.10	0.22 ± 0.15	0.13 ± 0.14	–	–	–
Peak pressure drift	mmHg·min^−1^	−0.3 ± 0.5	−4.3 ± 2.1	−3.0 ± 0.8	−3.0 ± 1.1	33.892	<0.001	0.693
	%LOP·min^−1^	−0.2 ± 0.2	−2.2 ± 1.2	−1.6 ± 0.4	−1.5 ± 0.6	30.797	<0.001	0.687
	*R* ^2^	0.05 ± 0.06	0.68 ± 0.25	0.77 ± 0.17	0.64 ± 0.19	–	–	–
Valley pressure drift	mmHg·min^−1^	0.4 ± 0.5	−1.9 ± 0.9	−1.8 ± 0.6	−1.3 ± 1.1	36.809	<0.001	0.710
	%LOP·min^−1^	0.2 ± 0.2	−1.0 ± 0.5	−1.0 ± 0.3	−0.7 ± 0.5	41.853	<0.001	0.736
	*R* ^2^	0.12 ± 0.11	0.48 ± 0.27	0.59 ± 0.20	0.40 ± 0.27	–	–	–

Abbreviations: LOP, limb occlusion pressure; *η*_p_^2^, partial eta squared.

Tourniquet cuff pressure peaks declined by ∼2–4 mmHg·min^−1^ (∼1%–2% LOP·min^−1^) across the BFR application period within the Saga, SmartCuffs, and Suji BFR devices. Tourniquet cuff pressure valleys declined by ∼1–2 mmHg·min^−1^ (∼1% LOP·min^−1^) across the BFR application period for the same BFR devices. The relationship between tourniquet cuff pressure peaks and valleys with respect to time was moderate-to-strong (*R*^2^ = 0.64–0.77) and weak-to-moderate (*R*^2^ = 0.40–0.59), respectively, in these BFR devices. The Delfi PTS for BFR exhibited trivial drifts in pressure peaks and valleys that had no relation with BFR application time (*R*^2^ ≤ 0.12). Individual data and pairwise comparisons can be found in the Supplementary Excel File (sheet: “Drift %LOP” and “Drift mmHg”).

## Discussion

The present report examined the ability of four commercial BFR devices (Delfi PTS for BFR, Saga, SmartCuffs, and Suji) to maintain and regulate tourniquet cuff pressure at the target pressure (i.e., 80% of the measured LOP) during BFR leg press exercise. The frequency distribution of pressures, as seen in Figure 1 and Supplementary Figures S1–S8, provides valuable insight regarding what pressures were occurring within the tourniquet cuff for each participant using each BFR device. A central finding was that the distribution of tourniquet cuff pressures can be highly variability within- and between-individuals, the degree of which appears dependent on the BFR device. In some devices, it was common to see the target pressure being poorly represented by the actual pressure within the tourniquet cuff during both “dynamic” (exercise) and “passive” (rest) BFR applications. For instance, during the exercise periods, the median percentage of time pressure was within ±10% the target pressure (i.e., 70%–90% LOP) was 95% (IQR: 93%–99%) for the Delfi PTS for BFR, whilst only 25% (IQR: 14%–52%) for Saga, 26% (IQR: 20%–62%) for SmartCuffs, and 34% (IQR: 30%–44%) for Suji. It was interesting to find that the Saga, SmartCuffs, and Suji BFR device tourniquets still had ∼20%–30% of pressure samples outside the largest pressure range examined: ±20% (i.e., 60%–100% LOP), during the BFR exercise periods.

Tourniquet cuff pressure during the rest periods was far more stable relative to the exercise periods as seen in the frequency distribution of pressures (Figure 1). However, a key finding was that despite pressure being kept within a narrower range, the percentage of LOP at which this occurred was, in some BFR devices, considerably different to the target pressure and highly variable between users. For instance, the median percentage of time pressure was within ±5% the target pressure (i.e., 75%–85% LOP) during the rest periods was 99% (IQR: 98%–99%) for the Delfi PTS for BFR, and 0% (IQR: 0%–2%) for the Saga, SmartCuffs, and Suji BFR devices. Describing the prescribed tourniquet cuff pressure as a constant (e.g., 80% of the measured LOP) makes an underlying assumption that the actual tourniquet cuff pressure is regulated at, or close to, the target pressure; the present data indicates that the accuracy of this assumption can be highly dependent on the BFR system used during both “dynamic” (exercise) and “passive” (rest) BFR applications.

Tourniquet cuff pressure was observed to be rising and falling during each exercise repetition (e.g., Figure 3) likely due to changes in limb circumference due to muscle contraction/relaxation as has been previously suggested (9). The magnitude of the pressure peaks and valleys varied between BFR devices: Delfi PTS for BFR (89 ± 2% and 72 ± 3% LOP), Saga (79 ± 9 and 58 ± 7% LOP), SmartCuffs (79 ± 9% and 61 ± 7% LOP), and Suji (90 ± 15 and 65 ± 10% LOP). Some participants using the Saga, SmartCuffs, and Suji BFR devices experienced pressure peaks exceeding 100% LOP and pressure valleys below 60% LOP (Figure 4), emphasising how far tourniquet cuff pressure can depart from the target pressure when BFR is applied during tasks requiring muscle contraction(s). Modern BFR pneumatic control systems, therefore, cannot yet completely mitigate the occurrence of pressure peaks/valleys during muscle contractions, however, the Delfi PTS for BFR shows that it is possible to attenuate them to within a magnitude of approximately ±10% the target pressure.

Pressures exceeding 100% LOP were observed in several cases for the Saga, SmartCuffs, and Suji BFR devices (Table 3). For instance, six participants were found to be exercising with pressures >100% LOP for ∼30%–55% of the time during the first exercise set using the Suji BFR device. Though it may seem reasonable to arrive at the conclusion that pressures >100% LOP would collapse the arterial wall and prevent arterial blood flow distal to the tourniquet, it is worth highlighting that current practices prescribe tourniquet cuff pressure relative to LOP measured at rest. Data from Barnett et al. found LOP in the upper-limb increased following low-intensity resistance exercise (138 ± 15 mmHg [rest]; 169 ± 20 mmHg [post-exercise), with arm circumference and brachial systolic blood pressure accounting for 69% of the variability in LOP (14). Under the assumption that LOP increases as a function of time during exercise, the actual degree of limb occlusion for a given pressure would, therefore, be lower relative to that under resting conditions. How much lower remains unclear without measuring LOP at various timepoints throughout the exercise period, however, this in itself would provide valuable information as to the degree of arterial occlusion actually being imposed in contexts where LOP is inclined to change from that when at rest. For the above reason, it thus remains to be verified whether complete arterial occlusions are indeed occurring when cuff pressure is found to exceed 100% of the measured LOP. An additional point to note is that LOP was measured automatically by the BFR device employed for the given BFR session, and not via Doppler ultrasound. It is, therefore, important that readers are made aware of the studies that have examined the reliability and/or validity of the LOP measurement provided by the Delfi PTS for BFR (11, 15, 16), Saga—The BFR Cuffs (17) (note—the study provided by Saga upon email request uses Airband's manufactured by Vald), and Smart Tools SmartCuffs 3.0 (18). The average difference between LOP-Doppler for the Smart Tools device was 8 ± 21 mmHg on the upper extremity and 11 ± 39 mmHg on the lower extremity. The average difference for the Vald device was 4 ± 14 mmHg on the lower extremity. This is compared to the average difference of the Delfi device of 1 ± 8 mmHg on the upper extremity and −1 ± 13 mmHg on the lower extremity. While there is not a statistically significant difference between automatic and doppler measurements of these devices, there is limited discussion or indication as to why some devices report greater differences than others.

Progressive loss of tourniquet cuff pressure occurred in the Saga, SmartCuffs, and Suji BFR systems by an average of ∼2–3 mmHg·min^−1^ (∼1%–2% LOP·min^−1^) throughout the entire BFR application period. At these rates, tourniquet cuff pressure would be expected to decline, on average, by ∼10–15 mmHg (∼5%–10% LOP) by the end of a typical BFR resistance exercise protocol lasting just 5 min. The Delfi PTS for BFR displayed trivial pressure loss throughout the BFR application period (<0.4 mmHg·min^−1^ and ≤0.2% LOP·min^−1^). Use of BFR devices that exhibit a progressive loss of pressure theoretically furthers the mismatch between the target and applied tourniquet cuff pressure. This is particularly so when considering the notion that not only is pressure being lost, but that LOP is also simultaneously increasing during exercise (14). The time-course of BFR pressure drifts remain unclear, such as whether they are continual, dampen over time, or eventually plateau. Pressure drifts highlight another important factor that can cause differences in tourniquet cuff pressure during BFR applications a degree of caution should be expressed when using prolonged BFR applications using BFR devices either with known susceptibility to pressure loss or that have not been verified to maintain the target pressure.

In an ideal scenario, BFR devices should maintain and regulate tourniquet cuff pressure at the target pressure with high consistency between-individuals. Figure 1 highlights that presently no BFR system can do such a task perfectly, and that some BFR systems seem more adept at doing so than others through the use of regulating pressure control. Further investigations should look to examine the test re-test reliability of tourniquet cuff pressure during BFR applications. Only one target pressure was investigated in the present investigation (80% LOP), future works is needed to examine whether dynamic and passive BFR applications at different target pressures (e.g., from 40%–80% LOP) cause simple rightward/leftward shifts in the pressure distribution or changes the shape of the distribution, which would have implications as to what range of pressures the limb is experiencing and how often. Similarly, only one exercise protocol was examined in the present study, and future studies should examine the effect of programmable exercise parameters (e.g., mode, intensity, repetition velocity, range of motion, etc.) of tourniquet cuff pressure variability. Lastly, it would be valuable to examine interface pressure which can provide useful insight into how the pressure within the tourniquet cuff is distributed around the limb.

Standardisation of the BFR pressure stimulus is of crucial importance to researchers, clinicians, and practitioners; therefore, one must be critical of whether certain BFR devices can continuously deliver tourniquet cuff pressure close to the prescribed pressure. There are several issues with BFR devices that provide inconsistent tourniquet cuff pressures within a group of individuals despite being prescribed at the same relative pressure (i.e., 80% of the measured LOP). Firstly, the safety, efficacy, and tolerability of BFR therapy has been linked to tourniquet cuff pressure (1, 7, 8). Secondly, applying a standardised BFR intervention in a group of individuals using a BFR device unable to accurately maintain and regulate tourniquet cuff pressure within- and/or between-individuals would likely increase variability in measured responses influenced by tourniquet cuff pressure. Thirdly, it is difficult to draw meaningful comparisons between studies due to the level of uncertainty that the BFR system employed actually maintained and/or regulated the tourniquet cuff pressure at, or even near, the target pressure. When tourniquet cuff pressure is a primary variable of interest in studies or reviews (e.g., comparing the effects of BFR at 60% vs. 80% LOP) this becomes a particular issue as the tourniquet cuff pressure is assumed to be constant.

A central question must be raised: what tourniquet cuff pressures have researchers and practitioners actually been applying? Without directly measuring tourniquet cuff pressure for each BFR application, the answer to such a question unfortunately remains elusive. As identified in the present analysis, this can be a particular issue for BFR devices that do not appear to provide a consistent and accurate pressure within narrow limits of the target pressure for all individuals. Unless a BFR device can be verified to impose consistent BFR pressures close to the target pressure with minimal variability within- and between-users, perhaps a greater level of caution must be exercised when interpreting BFR literature. The most logical way to avoid making assumptions about the applied tourniquet cuff pressure would be to measure the tourniquet cuff pressure *in-situ* for each BFR application. Manufacturers of BFR devices, therefore, should offer the capability to record tourniquet cuff pressure and ideally also limb interface pressure such that the raw pressure data can be downloaded and reported (e.g., as histograms). The impact and significance of short-term variations in tourniquet cuff pressure, in terms of the effect on acute and chronic outcomes, are not yet clear and further investigations are required to determine whether refined pneumatic pressure regulation might be of significant value.

## Data Availability

Original (raw) datasets and MATLAB code used to analyse them are available in the publicly accessible repository Zenodo: https://doi.org/10.5281/zenodo.14843857.
