# Supplementary material for: Tourniquet cuff pressure during blood flow restriction exercise
Source: Front Sports Act Living. 2025 Sep 2;7:1582387. doi: 10.3389/fspor.2025.1582387 (PMC12441828; doi:10.3389/fspor.2025.1582387)
Supplement: Supplementary file 2 [file Supplementaryfile1.docx]

Supplementary Figures and Tables

## Supplementary Tables

Supplementary Table 1. Blood flow restriction (BFR) device properties

| BFR Instrument Apparatus Capabilities | | | | |
| --- | --- | --- | --- | --- |
| Manufacturer and model | Delfi Medical – Personalized Tourniquet System for BFR | Saga - The BFR Cuffs | Smart Tools - SmartCuffs 3.0 | Suji Generation 1.0 |
| Method of pressure measurement | Automatic LOP measurement via cuff | Automatic personalized pressure measurement via cuff | Automatic personalized pressure measurement via cuff | Automatic personalized pressure measurement via cuff |
| Pressure regulation | 20 – 350 mmHg  ± 6 mmHg of set-point (10-second average under non-transient conditions without external leaks) | 250 mmHg maximum  No specification for regulation identified.  Note: maximum prescribed pressure (80% LOP) = 224mmHg (equivalent to 280mmHg LOP) | No regulation specifications identified.  Operating LOP tolerance +/- 15 mmHg | No specifications identified |
| Validity and reliability of limb occlusion pressure measurement | **References:**  Masri, B.A., Day, B., Younger, A.S. and Jeyasurya, J., 2016. Technique for measuring limb occlusion pressure that facilitates personalized tourniquet systems: a randomized trial. *Journal of Medical and Biological Engineering*, *36*, pp.644-650.  Hughes, L., McEwen, J. 2021. Investigation of clinically acceptable agreement between two methods of automatic measurement of limb occlusion pressure: a randomized trial. BMC Biomedical Engineering, 3, pp. 1-8.  Hughes, L., Jeffries, O., Waldron, M., Rosenblatt, B., Gissane, C., Paton, B. and Patterson, S.D., 2018. Influence and reliability of lower-limb arterial occlusion pressure at different body positions. *PeerJ*, *6*, p.e4697.  McEwen USRE50013 Tourniquet system for personalized restriction of blood flow  McEwen EP3171794 Tourniquet system for personalized restriction of blood flow | **References:**  Zhang, W.Y., Zhuang, S.C., Chen, Y.M. and Wang, H.N., 2024. Validity and reliability of a wearable blood flow restriction training device for arterial occlusion pressure assessment. *Frontiers in Physiology*, *15*, p.1404247.  Note: This study was provided by Saga upon email request. The study was completed with a device from a different BFR manufacturer (AirBands by Vald). | **References:**  Abbas, M.J., Dancy, M.E., Marigi, E.M., Khalil, L.S., Jildeh, T.R., Buckley, P.J., Gillett, J., Burgos, W. and Okoroha, K.R., 2022. An automated technique for the measurement of limb occlusion pressure during blood flow restriction therapy is equivalent to previous gold standard. *Arthroscopy, Sports Medicine, and Rehabilitation*, *4*(3), pp.e1127-e1132. | No response to an email request within five working days.  No literature identified. |
| Tourniquet Cuff Properties | | | | |
| Manufacturer and model | Delfi Medical Easi-Fit BFR Cuff and Matching Limb Protection Sleeve | Saga - The BFR Cuffs | Smart Tools - SmartCuffs 3.0 | Suji Generation 1.0 |
| Cuff selection | Limb size chart by manufacturer | Limb size chart by manufacturer | Limb size chart by manufacturer | Limb size chart by manufacturer |
| Cuff width (width x length) | 24” x 4.5”  34” x 4.5” | Leg Regular - 30.75” x 4” | Medium – 23.25” x 4.25”  Large – 34” x 4.25” | Leg Cuff (30” x 4”) |
| Material | Information not available | Information not available | Information not available | Information not available |
| Type of internal bladder system | Single bladder system -  Fully encircles the limb | Single bladder system – bladder does not fully encircle the limb | Single bladder system -  Fully encircles the limb | Single bladder system -  Fully encircles the limb |
| Cuff shape | Contoured | Cylindrical | Cylindrical | Cylindrical |
| Internal bladder length | 24 – 21.5”  34 - 31.5” | 12.75” | Medium - 22.5”  Large - 33” | 29” |
| BFR Pressure Prescription | | | | |
| Limb occlusion pressure (mmHg) (n = 15) | 155 ± 15 | 191 ± 34 | 157 ± 13 | 135 ± 11 |
| Posture used for measurement of limb occlusion pressure | Supine. Participants were instructed to lay still and quiet throughout the entire duration of the measurement. | | | |
| Timings and pressure application | Inflated ~5-10-seconds pre-exercise and deflated ~5-10-seconds post-exercise. Applied continuously throughout the exercise and rest periods (~6.5 minutes). | | | |
| Target vs actual pressure applied | As per the findings of the present study. | | | |

Abbreviations: LOP, limb occlusion pressure.

## Supplementary Figures


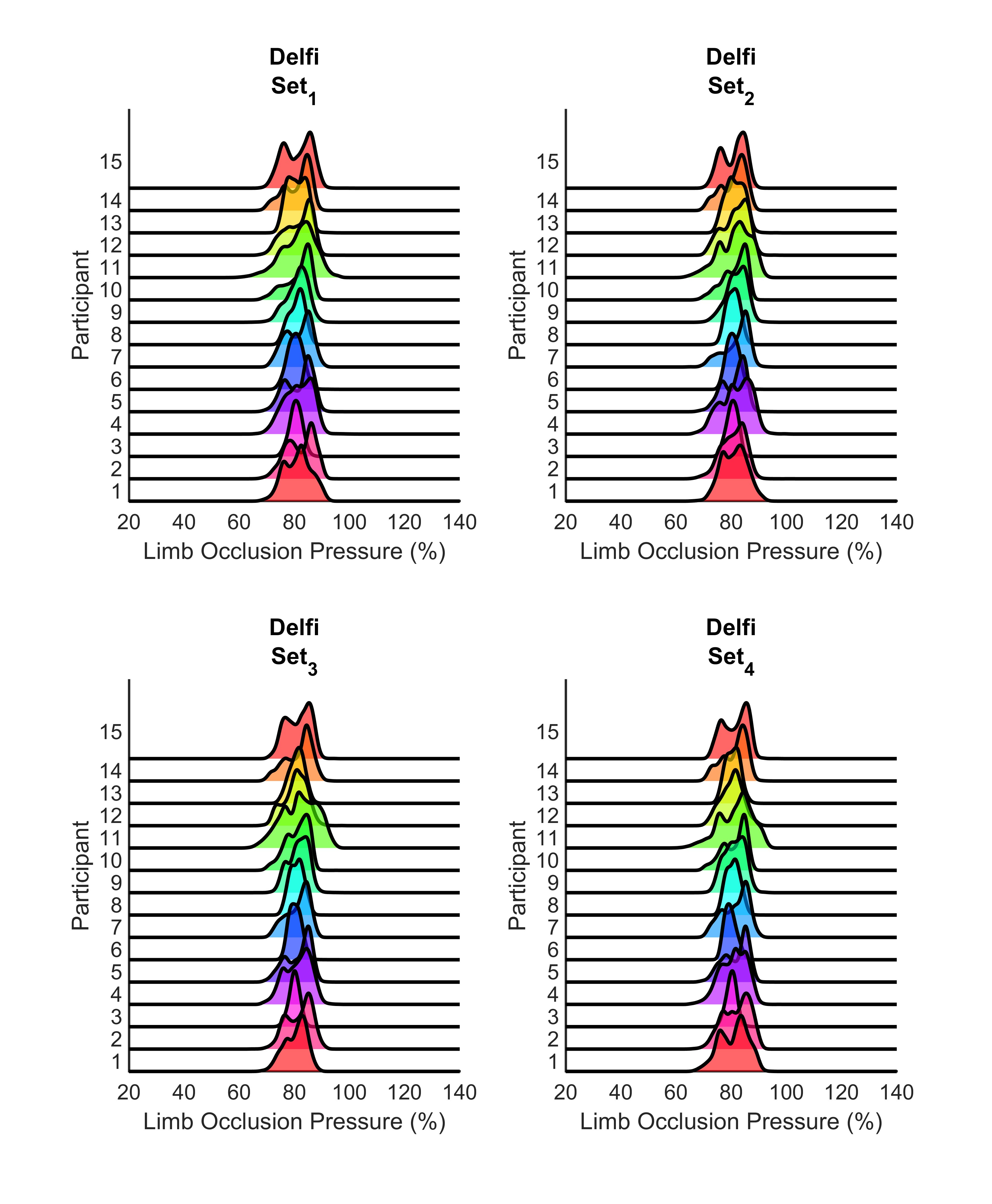


Supplementary Figure 1. Distribution of tourniquet cuff pressure during each exercise set for the Delfi Personalized Tourniquet System for Blood Flow Restriction.

Supplementary Figure 2. Distribution of tourniquet cuff pressure during each rest period for the Delfi Personalized Tourniquet System for Blood Flow Restriction.


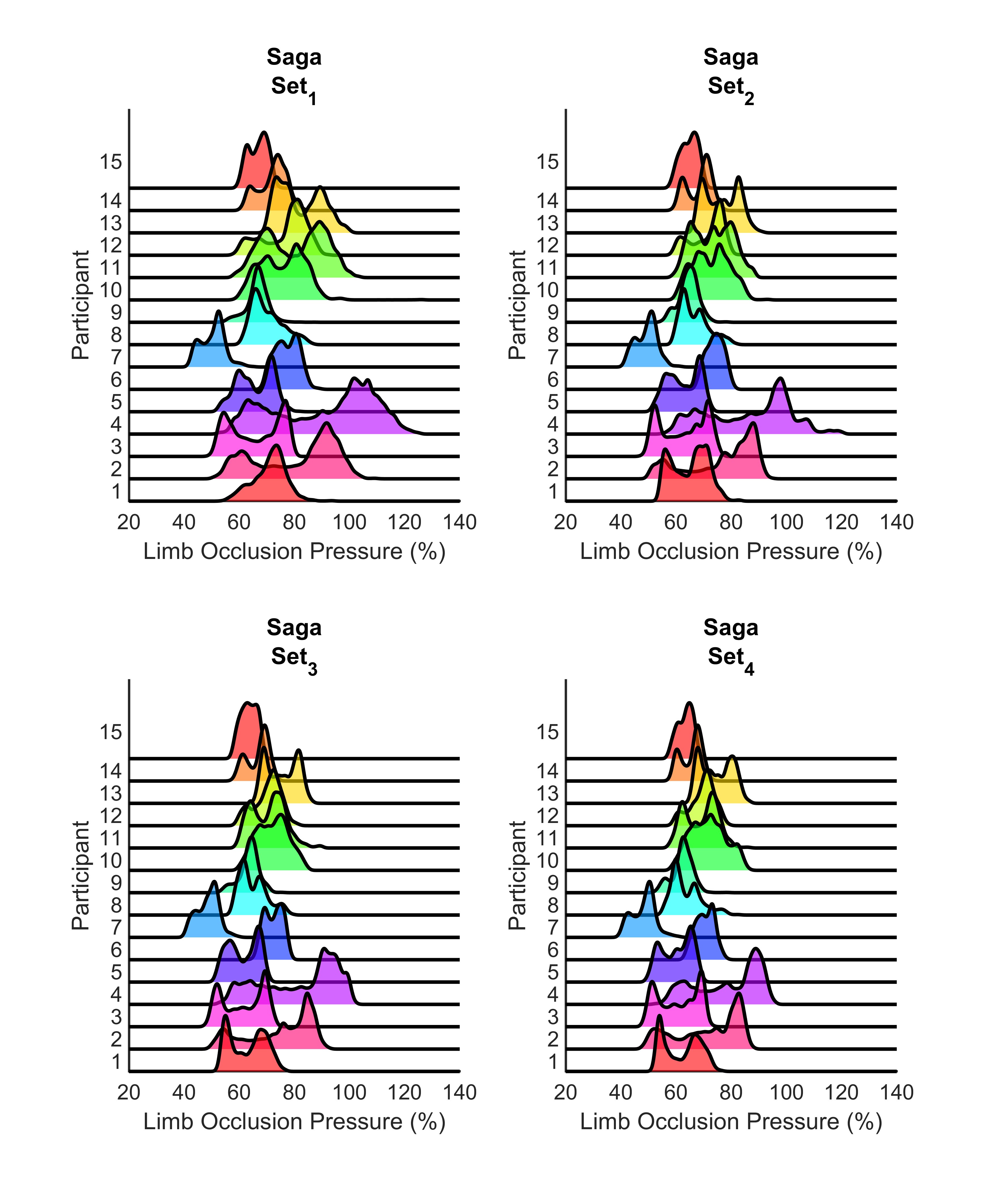


Supplementary Figure 3. Distribution of tourniquet cuff pressure during each exercise set for the Saga BFR device.

Supplementary Figure 4. Distribution of tourniquet cuff pressure during each rest period for the Saga BFR device.


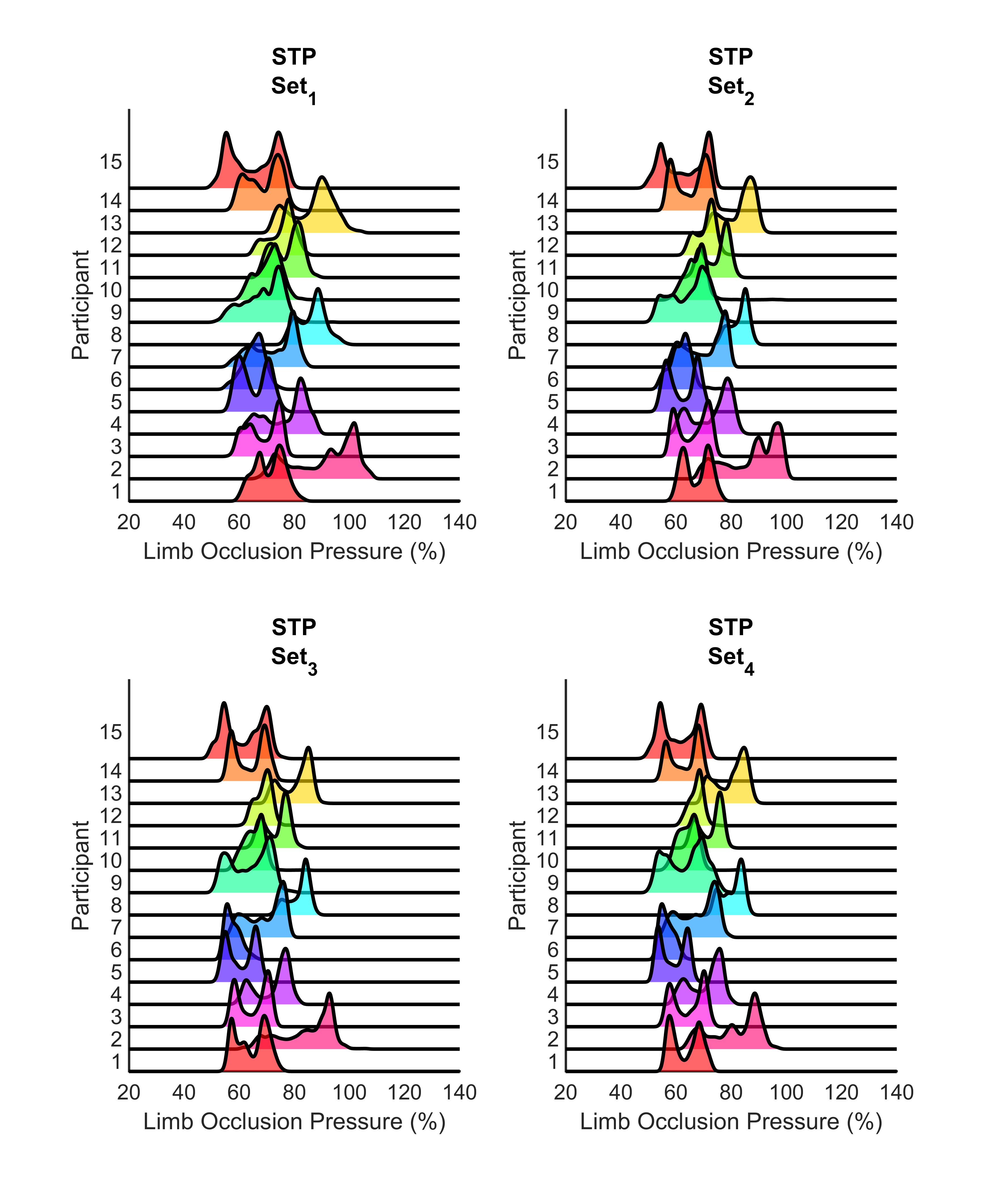


Supplementary Figure 5. Distribution of tourniquet cuff pressure during each exercise set for the SmartTools (STP) BFR device (SmartCuffs).

**Supplementary Figure 6.** Distribution of tourniquet cuff pressure during each rest period for the SmartTools (STP) BFR device (SmartCuffs).


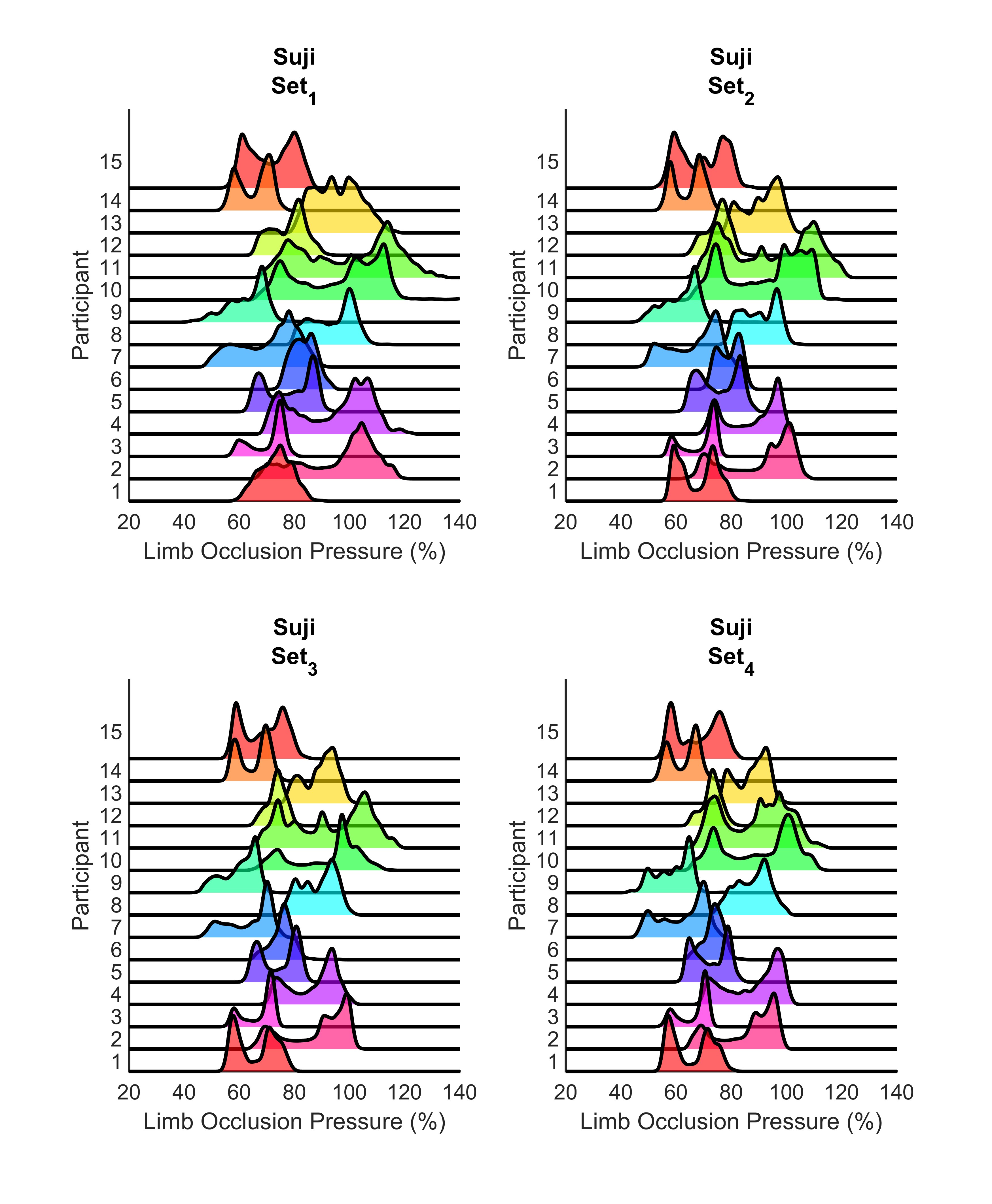


Supplementary Figure 7. Distribution of tourniquet cuff pressure during each exercise set for the Suji BFR device.

Supplementary Figure 8. Distribution of tourniquet cuff pressure during each rest period for the Suji BFR device
